# Genomics, evolution, and crystal structure of a new family of bacterial spore kinases

**DOI:** 10.1002/prot.22663

**Published:** 2009-11-24

**Authors:** Eric D Scheeff, Herbert L Axelrod, Mitchell D Miller, Hsiu-Ju Chiu, Ashley M Deacon, Ian A Wilson, Gerard Manning

**Affiliations:** 1 Razavi Newman Center for Bioinformatics, Salk Institute for Biological StudiesLa Jolla, California; 2 Joint Center for Structural Genomics (JCSG)Menlo Park, California; 3 Stanford Synchrotron Radiation Lightsource, SLAC National Accelerator LaboratoryMenlo Park, California; 4 Department of Molecular Biology, The Scripps Research InstituteLa Jolla, California

**Keywords:** protein kinase-like, PKL, CAK, endospore, pseudokinase, YtaA, CotS, YutH, YsxE, BSK

## Abstract

Bacterial spore formation is a complex process of fundamental relevance to biology and human disease. The spore coat structure is complex and poorly understood, and the roles of many of the protein components remain unclear. We describe a new family of spore coat proteins, the bacterial spore kinases (BSKs), and the first crystal structure of a BSK, YtaA (CotI) from *Bacillus subtilis*. BSKs are widely distributed in spore-forming *Bacillus* and *Clostridium* species, and have a dynamic evolutionary history. Sequence and structure analyses indicate that the BSKs are CAKs, a prevalent group of small molecule kinases in bacteria that is distantly related to the eukaryotic protein kinases. YtaA has substantial structural similarity to CAKs, but also displays distinctive features that broaden our understanding of the CAK group. Evolutionary constraint analysis of the protein surfaces indicates that members of the BSK family have distinct clade-conserved patterns in the substrate binding region, and probably bind and phosphorylate distinct targets. Several classes of BSKs have apparently independently lost catalytic activity to become pseudokinases, indicating that the family also has a major noncatalytic function. Proteins 2010. © 2009 Wiley-Liss, Inc.

## INTRODUCTION

Many Gram-positive bacteria form endospores in response to stress. Spores are highly resistant to destructive agents such as heat, chemicals, and radiation, and can persist in harsh environments for many years.^1^ Although highly stable, spores can rapidly germinate when conditions become hospitable.^2^ The process of sporulation serves as a model for regulation in bacteria,^3^ and is important to the pathogenesis of species such as *Bacillus anthracis* and *Clostridium botulinum*.^4^

Bacterial spores have a layered structure which includes a protective protein coat. The coat must exclude harmful agents, while also allowing nutrients to enter to trigger germination.^2^ It is a complex structure, containing at least 70 different proteins in *Bacillus subtilis*.^1^ The roles of many coat proteins are poorly understood, but some are enzymes with known roles,^5^,^6^ or have sequence similarity to enzymes.^1^

YtaA (CotI) of *B. subtilis* is a member of a family of proteins specific to the phylum *Firmicutes*, which are implicated in spore formation and often form part of the spore coat.^7^–^9^ Apart from a brief mention of some members in InterPro,^10^ it has not been reported that these proteins form a single family, or that they are kinase homologues. We have therefore named this family the bacterial spore kinases (BSKs), and have carried out a comprehensive genomic and evolutionary analysis of the family, coupled to a combined analysis of sequence conservation and the crystal structure of YtaA.

The BSKs constitute a new family within the CAK kinases.^11^ CAKs adopt a protein kinase-like (PKL) fold, with distinctive CAK-specific structural elements.^12^ They usually phosphorylate small molecules, and are named for the **c**holine and **a**minoglycoside **k**inase members, which were the first structures to be described.^13^,^14^ Although all CAKs share a similar fold, they span a wide sequence and phylogenetic space.^11^ Beyond the value to our understanding of sporulation, the structure of YtaA provides new insights into the evolution of the PKL superfamily. While similar to other CAKs, YtaA also displays distinctive changes, exemplifying the array of innovations that have taken place in the PKL fold over long evolutionary timescales.

## MATERIALS AND METHODS

### Crystallization

YtaA was crystallized using the nanodroplet vapor diffusion method^15^ with standard JCSG crystallization protocols.^16^ Screening for diffraction was carried out using the Stanford Automated Mounting system^17^ at the Stanford Synchrotron Radiation Laboratory (SSRL, Menlo Park, CA). The crystallization reagent that produced the YtaA crystal used for structure determination consisted of 2.23*M* ammonium sulfate, 0.1*M* citric acid pH 5.57. Ethylene glycol was added as a cryoprotectant to a final concentration of 15% (v/v). The YtaA crystal was indexed in hexagonal space group P6_4_22 (Table I).^18^,^19^

**Table I:** Summary of Crystal Parameters, Data Collection, and Refinement Statistics for YtaA (PDB ID: 2Q83)

Space group	P6_4_22		
Unit cell parameters	*a* = *b* = 173.00, *c* = 192.57 Å		
Data collection	λ_1_ MAD-Se	λ_2_ MAD-Se	λ_3_ MAD-Se
Wavelength (Å)	0.9116	0.9792	0.9791
Resolution range (Å)	29.96–2.50	29.96–2.50	29.99–2.71
Number of observations	639,503	631,260	511,467
Number of unique reflections	59,155	59,113	46,847
Completeness (%)	99.9 (100.0)^a^	99.9 (100.0)	99.9 (100.0)
Mean *I*/σ(*I*)	17.0 (3.0)^a^	17.2 (2.5)	14.2 (1.9)
*R*_sym_ on *I* (%)	12.6 (86.4)^a^	12.5 (86.9)	16.9 (1.398)
Highest resolution shell (Å)	2.56–2.50	2.56–2.50	2.78–2.71
Model and refinement statistics			
Resolution range (Å)	29.96–2.50	Dataset used in refinement	λ_1_ MADSe
Number of reflections (total)	59,108^b^	Cutoff criteria	|*F*| > 0
Number of reflections (test)	2988	*R*_cryst_	0.198
Completeness (% total)	100.0	*R*_free_	0.210
Stereochemical parameters			
Restraints (RMS observed)			
Bond length (Å)	0.012		
Bond angle (°)	1.66		
Average isotropic *B*-value (Å2)	40.7		
ESU based on *R*_free_ value (Å)	0.169		
Protein residues/atoms	332/5615		
Water molecules	185		

a Highest resolution shell.

b Typically, the number of unique reflections used in refinement is slightly less than the total number that were integrated and scaled. Reflections are excluded due to systematic absences, negative intensities, and rounding errors in the resolution limits and cell parameters.

ESU, estimated overall coordinate error^18^,^19^; *R*_sym_, Σ|*I*_*i*_ – <*I*_*i*_>|/Σ|*I*_*i*_|, where *I*_*i*_ is the scaled intensity of the *i*th measurement and <*I*_*i*_> is the mean intensity for that reflection; *R*_cryst_, Σ| |*F*_obs_| – |*F*_calc_| |/Σ|*F*_obs_|, where *F*_calc_ and *F*_obs_ are the calculated and observed structure factor amplitudes, respectively; *R*_free_, as for *R*_cryst_, but for 4.9% of the total reflections chosen at random and omitted from refinement.

### Data collection, structure solution, and refinement

Multiple-wavelength anomalous diffraction (MAD) data were collected at the SSRL on beamline BL11-1 at wavelengths corresponding to the high-energy remote, (λ_1_), inflection (λ_2_), and peak (λ_3_) of a selenium MAD experiment. The datasets were collected at 100 K with a MarMosaic 325-mm CCD detector using Blu-Ice.^17^ The MAD data were integrated and reduced using MOSFLM^20^ and then scaled with the program SCALA.^18^ The selenium substructure solution and phasing were performed with SHELXD^21^ and SOLVE,^22^ and automatic model building was performed with iterative RESOLVE.^23^ Model completion and refinement were performed with Coot^24^ and REFMAC 5.2^25^ using the remote (λ_1_) dataset. Data and refinement statistics for YtaA are summarized in Table I.

### Validation and deposition

Analysis of the stereochemical quality of the model was accomplished using AutoDepInputTool,^26^ MolProbity,^27^ SFcheck 4.0,^18^ and WHATIF 5.0.^28^ Atomic coordinates and experimental structure factors of YtaA have been deposited in the PDB^29^ under the code 2Q83.

### Structure analysis

Coordinates for structures other than YtaA were collected from the PDB^29^ as follows: homoserine kinase 2 (HSK2, PDB ID: 2PPQ), choline kinase (ChoK, PDB ID: 1CKP), aminoglycoside phosphotransferase (APH, PDB ID: 1L8T), and protein kinase A (PKA, PDB ID: 1CDK). MolProbity^27^ was used to add optimized hydrogen atoms; all suggested Asn/Gln/His flips were also accepted. Structural alignments were made with DaliLite.^30^

### Sequence analysis

Sequence homologs of YtaA were gathered using BLAST and HMM searches of the NCBI peptide nonredundant database^31^ and the IMG microbial genome database.^32^ Chromosomal clustering and operon structure was verified using IMG. Sequences from the BSK family were aligned with MUSCLE^33^ followed by manual curation, using the YtaA structure to determine appropriate gap locations. A nonredundant alignment was made by removing sequences from strain variants. A representative alignment of HSK2 sequences was made from sequences in Kannan et al.,^11^ filtered to retain sequences with <80% identity with cd-hit,^34^ followed by alignment with MUSCLE. The BSK and HSK2 alignments were merged by profile–profile alignment in ClustalX,^35^ and then manually edited to maximize agreement with the DaliLite alignment of YtaA and HSK2 structures (Supporting Information Figure S1). Sequence motif logos were made with the WebLogo server.^36^ Evolutionary constraints were mapped to the YtaA structure using the ConSurf server,^37^ using appropriate sections of the alignment as input. Conservation scores were calculated with the default Bayesian method, and positions that scored within the top 3 conservation bins in ConSurf were reported as conserved.

### Phylogenetic analysis

The BSK/HSK2 family alignment was edited to remove sparsely populated (uninformative) columns and partial sequences (Supporting Information Figure S2). The alignment was evaluated with PHYML^38^ using the following settings: LG substitution model, four substitution categories, estimated gamma shape parameter, optimization of topology/branch length/substitution rate parameters, (the slower but more accurate) SPR tree topology search method, and 100 bootstraps (Supporting Information Figure S3). Taxonomy is from Bergey's classification, based primarily on 16s rRNA.^39^

### Raw data availability

All supporting information is available (in its original file formats) at http://kinase.com/microbial/bsk.

## RESULTS AND DISCUSSION

### BSK: A new family of spore-associated kinases

**Figure 1:**
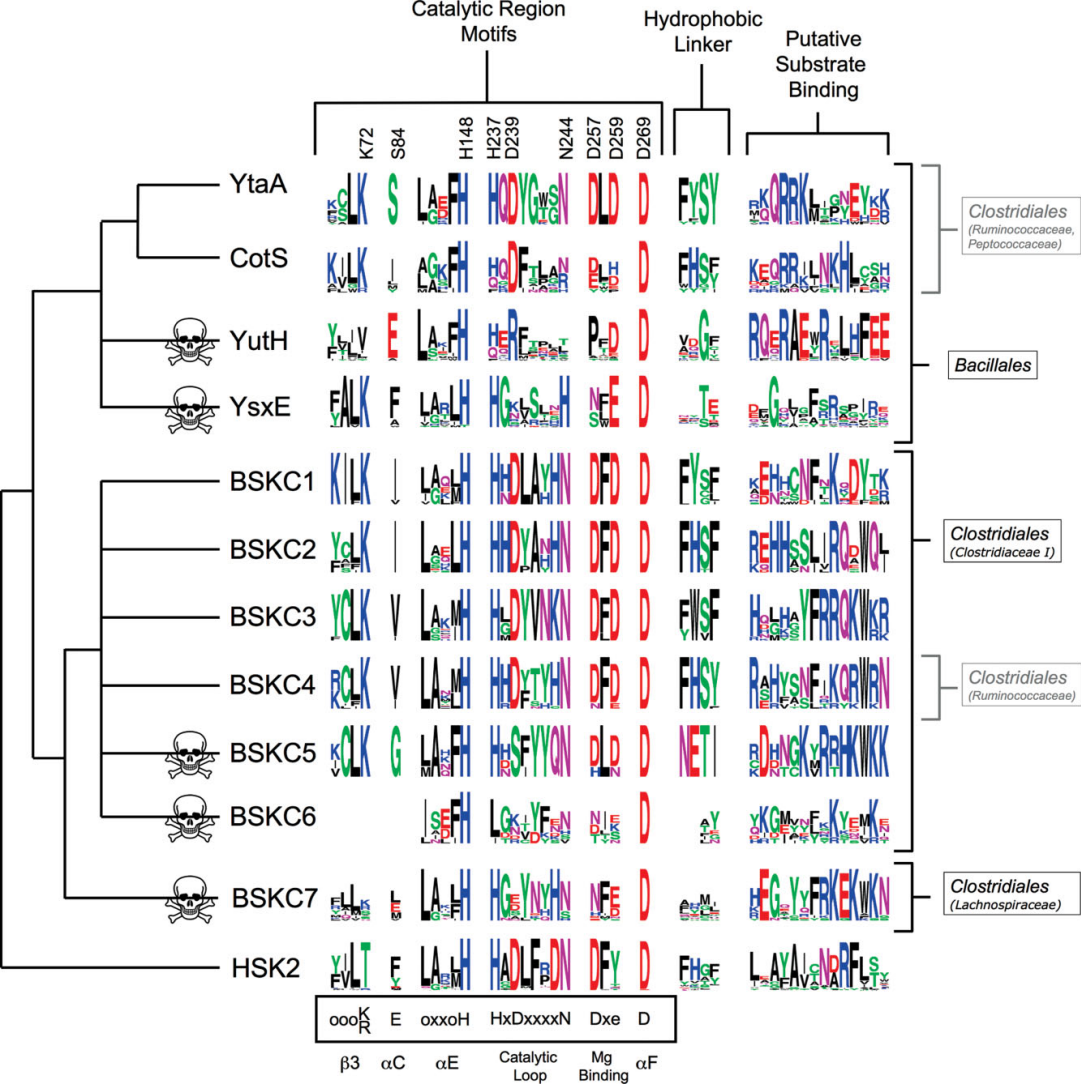
Conserved motifs in BSKs, arranged by proposed phylogeny. Logos show the relative conservation at selected positions within each of the major family members, with HSK2 as an outgroup. Key residue numbers in YtaA are labeled on top, and consensus CAK motifs and structural elements are labeled on the bottom (o = any hydrophobic residue; x = any residue; lowercase = partially conserved). Skull and crossbones indicate predicted pseudokinases; some CotS orthologs may also be pseudokinases. Structural residues such as H148 and D269 are highly conserved, whereas catalytic residues are lost in pseudokinases and many other positions are conserved but distinct between classes. The hydrophobic linker and putative substrate binding motifs are structural motifs that are discontinuous in the primary sequence, and are shown with the intervening sequence removed. The tree is a schematic, inferred from phylogenetic analysis (Supporting Information Figure S3) and species taxonomy (Supporting Information Table S1). The major taxonomic ranges of each BSK are shown on the right.

We gathered over 220 homologous sequences from public databases that form a distinct new family (BSK) within the CAK kinases. Homologs were from the phylum *Firmicutes*, mostly within spore-forming species in the orders *Bacillales* and *Clostridiales* and largely absent from nonsporulating species (Supporting Information Tables S1 and S2). Multiple BSKs exist in many species, with four predominant within the *Bacillales*, while in *Clostridiales* six distinct BSKs are found in *Clostridiaceae* and one in *Lachnospiraceae* (See Fig. 1).

Four BSKs are found in *B. subtilis* and many other *Bacillaceae*: YutH and YsxE are present in almost all spore-forming species, whereas YtaA and CotS are more restricted. All four are experimentally implicated in sporulation. *CotS* and *ytaA* share a common promoter, controlled by the spore-specific factors σ^K^ and GerE^7^–^9^,^40^,^41^ and both are packaged into the spore coat in a CotE-dependent manner.^42^ CotS is not detectable in vegetative cells,^8^ indicating that its role is spore-specific. YutH and YsxE are also packaged into the coat,^42^,^43^ and ysxE shares an operon with the spore coat protein SpoVID^44^; both are regulated by the spore-specific σ^E^.^9^ CotS mutants produce morphologically normal spores.^7^,^8^ Mutants lacking *yutH* or *ysxE* also produce spores that are morphologically normal, but more sensitive to lysozyme, hypochlorite, and predation,^45^ indicating that BSKs are evolutionarily important for spore survival in natural environments.

Six distinct BSKs (bacterial spore kinase Clostridiales, BSKC1–BSKC6) are found in members of the spore-forming genus *Clostridium* within the family *Clostridiaceae I* (the genus *Clostridium* is paraphyletic, with some members in other families within *Clostridiales*^39^). Expression profiling in *Clostridium acetobutylicum* shows that BSKC4 is selectively expressed during sporulation.^46^ Reanalysis of these data suggests that BSKC3 and BSKC5 may also be induced during sporulation, with BSKC3 having the stronger pattern. Accordingly, we find plausible conserved σ^E^ binding sites in the *BSKC4* and *BSKC3* promoters, a weakly conserved site in the *BSKC5* promoter, and no site in the single operon containing *BSKC1, 2*, and *6* (data not shown). This suggests that the expanded family in *Clostridium* may have diverged into spore-associated and nonspore-associated functions.

Most sporulating species within *Clostridiales* and *Bacillales* have BSKs and vice versa, but there are exceptions. A single gene, *BSKC7*, is present in the *Lachnospiraceae*, in both spore formers and nonspore formers (Supporting Information Table S1). Conversely, *Clostridium difficile* (family *Peptostreptococcaceae*^39^) forms spores but has no BSKs.

Sequence similarity between BSKs is low (highest pairwise identity is ∼30%), making phylogenetic reconstruction difficult, with low-bootstrap values at many basal branches (Supporting Information Figure S3). However, when coupled to known species relationships, our results suggest that the most parsimonious evolutionary scenario requires independent expansions in *Bacillales* and *Clostridiales* (see Fig. 1).

In addition to the highly represented BSKs, several divergent members are found in some species (Supporting Information Table S1). Most notable are four homologs seen within *Heliobacterium modesticaldum* (bacterial spore kinase Heliobacterium, BSKH1-4), an unusual phototrophic member of a distinct family in *Clostridiales*.^47^ Similarity is weak between the BSKHs and the BSKCs, suggesting that the BSKHs represent an independent expansion in *H. modesticaldum* that may be related to the shared sporulating phenotype (Supporting Information Figure S3).

Several sporulating species in *Clostridiales* may form a bridge between this order and *Bacillales*. *Clostridium thermocellum* contains YtaA, CotS, and BSKC4. *Symbiobacterium thermophilum* and *Desulfotomaculum acetoxidans* have no BSKCs, but have YtaA (*D. acetoxidans* also has CotS) (Supporting Information Table S1). Although horizontal transfer cannot be ruled out, *C. thermocellum* could represent an ancestral state, from which expansion in *ytaA* could produce the *Bacillales* genes, and expansion in *BSKC4* could produce *Clostridiales* genes.

### Several BSKs are predicted to be catalytically inactive

The sequence motifs required for enzymatic activity in PKL kinases have been extensively explored and mapped to the structure of PKA,^11^ the prototype of PKL kinases.^48^ Although the CAK family displays considerable plasticity in these motifs relative to other PKL families, a few key residues have remained nearly invariant, most notably D239^YtaA^ (D166^PKA^), which coordinates the target substrate hydroxyl group in substrate-bound structures,^49^,^50^ and is believed to be required for catalytic activity.^51^

Five BSKs have lost D239^YtaA^, along with other motifs generally required for enzymatic function, and we predict that they are pseudokinases.^52^ Assuming that their common ancestor was active, our evolutionary model indicates that BSKs lost catalytic activity independently in *Bacillales* and *Clostridiales*, and possibly in *Lachnospiraceae* (see Fig. 1). This pattern suggests a common nonenzymatic function for BSKs, coupled to a sometimes dispensable role as a kinase.

These five pseudokinase BSKs have a variety of inactivating mutations, in addition to the loss of D239^YtaA^ (see Fig. 1). Three eukaryotic protein kinase (ePK) pseudokinase structures have recently been published.^53^–^55^ All show a selective loss of catalytic residues, coupled with retention of residues required for folding, resulting in structures that are highly similar to their catalytically active relatives. A similar pattern is seen in the BSK pseudokinases, though the sequence changes that occur on inactivation are even more extreme. For example, despite the poor overall sequence similarity between BSKs, H148^YtaA^ (H158^PKA^) and D269^YtaA^ (D220^PKA^), which form hydrogen bonds to stabilize the fold of the C-terminal lobe,^12^ are conserved in almost all BSK pseudokinases, indicating that this interaction is critical for a family-wide function. However, H237^YtaA^ (H164^PKA^) which forms hydrogen bond interactions that directly stabilize the geometry of the active site,^12^ is only partially conserved in YutH and lost completely in BSKC6. In fact, BSKC6 has lost the entire N-terminal lobe of the kinase domain (see discussion of structure later), similar to some viral PKL kinases,^11^ and the KIND domain in metazoans.^56^ This change appears to be relatively recent: There are BSK6-like proteins (BSKC6L) with intact N-terminal lobes in *Caldicellulosiruptor saccharolyticus* and *Anaerocellum thermophilum* (Supporting Information Tables S1 and S3). BSKC6L appears to be the ancestral form of BSK6, though it still lacks residues required for enzymatic activity.

The remaining members of the BSK family display substantial selective conservation of known CAK catalytic motifs (though some aspects of the YtaA active site are unusual, see discussion later). This pattern strongly suggests that these proteins will be active kinase enzymes (see Fig. 1). However, no BSK has been experimentally assayed for catalytic activity, and we therefore define these members only as putatively active BSKs.

Interestingly, while CotS is a putatively active BSK, it may also be a pseudokinase in some species. Although it conserves D239^YtaA^, it frequently loses the DxD motif (Dxe across all CAKs, first position is D257^YtaA^) and N244^YtaA^ (see Fig. 1). These motifs are not universally required for PKL kinase activity, but they are almost completely conserved in putatively active BSKs. CotS also sometimes loses H237^YtaA^. Remarkably, three species outside the *Bacillaceae* (including *C. thermocellum*) have a CotS that retains all active site residues (Supporting Information Tables S1 and S3). Thus, as with BSKC6, we can directly observe an apparent ongoing process of loss of functionality within CotS through the examination of current genome sequences.

### YtaA and cotS form a conserved chromosomal cluster with genes involved in nucleotide sugar metabolism

**Figure 2:**
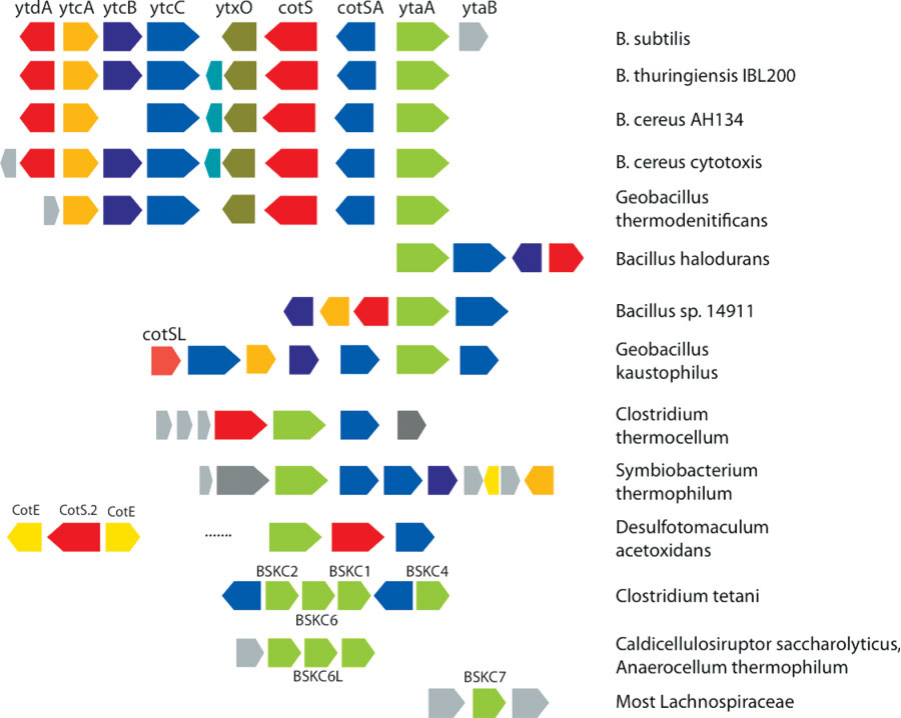
Conservation patterns near the *ytaA/cotS* loci. Despite considerable rearrangements, the *ytaA/cotS* genes are consistently colocated and coregulated with glycosyl transferases (*ytcC* and *cotSA*) and frequently with nucleotide sugar metabolizing genes (*ytdA* and *ytcA-B*). Genes are color-coded by orthology; gray represents genes neither conserved in the cluster nor spore-associated. Gene lengths are not to scale.

*YtaA* and *cotS* form a conserved chromosomal cluster with a pair of related glycosyl transferases (*cotSA* and *ytcC*; 47% sequence ID) and a set of enzymes involved in nucleotide sugar metabolism (*ytcA-B* and *ytdA*; Table II, Fig. 2). While the order and operon membership of these genes varies between species, their conserved tight clustering and predicted coregulation suggests functional interactions. Indeed, both CotSA and YtcC are found in the spore^41^ and CotSA requires CotS to be assembled into the spore,^58^ suggesting that they might be binding partners.

**Table II:** Genes in the Chromosomal Cluster Containing cotS and ytaA, with their COG Assignments^57^

Gene symbol	COG	Name	Reaction
ytdA	COG1210	UDP-glucose pyrophosphorylase	Glucose-1-phosphate + UTP → UDP-glucose
ytcA	COG1004	UDP-glucose 6-dehydrogenase	UDP-glucose + NAD → UDP-glucuronate + NADH
ytcB	COG0451	UDP-glucose epimerase	Interconverts UDP-glucose and UDP-galactose
cotSA, ytcC	COG0438	Spore-associated glycosyl transferase	Transfers NDP-sugars to protein or small molecule acceptors
cotS, ytaA	COG2344	BSK	Kinase (CotS may be inactive)

This linkage is further supported by coordinated gene loss in several species. No genome has a *ytaA/cotS* without this class of glycosyl transferase, or a glycosyl transferase without *ytaA* or *cotS* (Supporting Information Table S1). Our phylogenetic model suggests that there have been multiple coordinated losses of these genes, sometimes linked to losses of the *ytc* and *ytd* genes. Four of the 17 sequenced strains of *B. thuringiensis* have a chromosomal cluster containing both BSKs and both glycosyl transferases, *ytcB* and *ytdA*. The other 13 strains lack all six genes. Similarly, of six *Geobacillus* species, one (WCH70) lacks both BSKs and both glycosyl transferases, whereas another (Y412MC10) has lost one of each and both have also lost some of the *ytc/ytd* genes. Two of eight *B. cereus* species have both BSKs and both glycosyl transferases, and the rest lack both.

A BSK-glycosyl transferase link is also seen in most *Clostridium* species, where *BSKCs 2, 6*, and *1* are clustered in a single operon and *BSKC4* is nearby (see Fig. 2). This operon is flanked by two glycosyl transferases which are distantly related to *cotSA* and *ytcC*. By contrast, the conserved linkage of *BSKC7* in the mostly nonsporulating *Lachnospiraceae* is to unrelated genes.

### The crystal structure of YtaA indicates that BSKs are CAK kinases with unusual features

**Figure 3:**
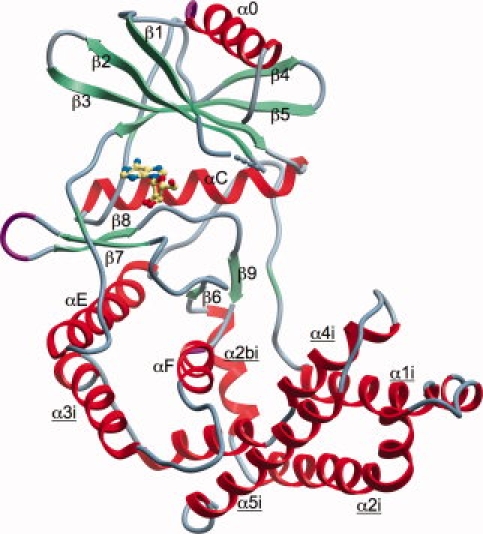
Crystal structure of YtaA, colored by secondary structure. CAK-specific elements are labeled with an underline.

The crystal structure of YtaA, at 2.5 Å resolution (Table I), has an overall similarity to previously determined CAK structures. YtaA contains the PKL bilobed fold, with a smaller, mostly β-stranded N-terminal lobe, and a larger, mostly α-helical C-terminal lobe (see Fig. 3).^12^ The electron density indicated that a molecule containing an adenosine moiety was bound in the interlobe cleft, where the adenosine moieties of ATP/ADP are found in other PKL structures. This molecule was present due to copurification with YtaA; it was not provided in the crystallization conditions (see “Materials and Methods” section). There was no electron density beyond the ribose of the adenosine moiety, and the ribose was partially disordered (Supporting Information Figure S4). Thus, it was unknown if the expected phosphate groups were genuinely absent or simply disordered. We therefore modeled the ligand as adenosine. In addition, the phosphate-binding loop between Q52 and A57 had poor electron density, and could not be reliably modeled. This loop normally interacts with the phosphates of the ATP and is assumed to be disordered. The N-terminus of the protein, G1-E21, was also not resolved in the electron density maps.

**Figure 4:**
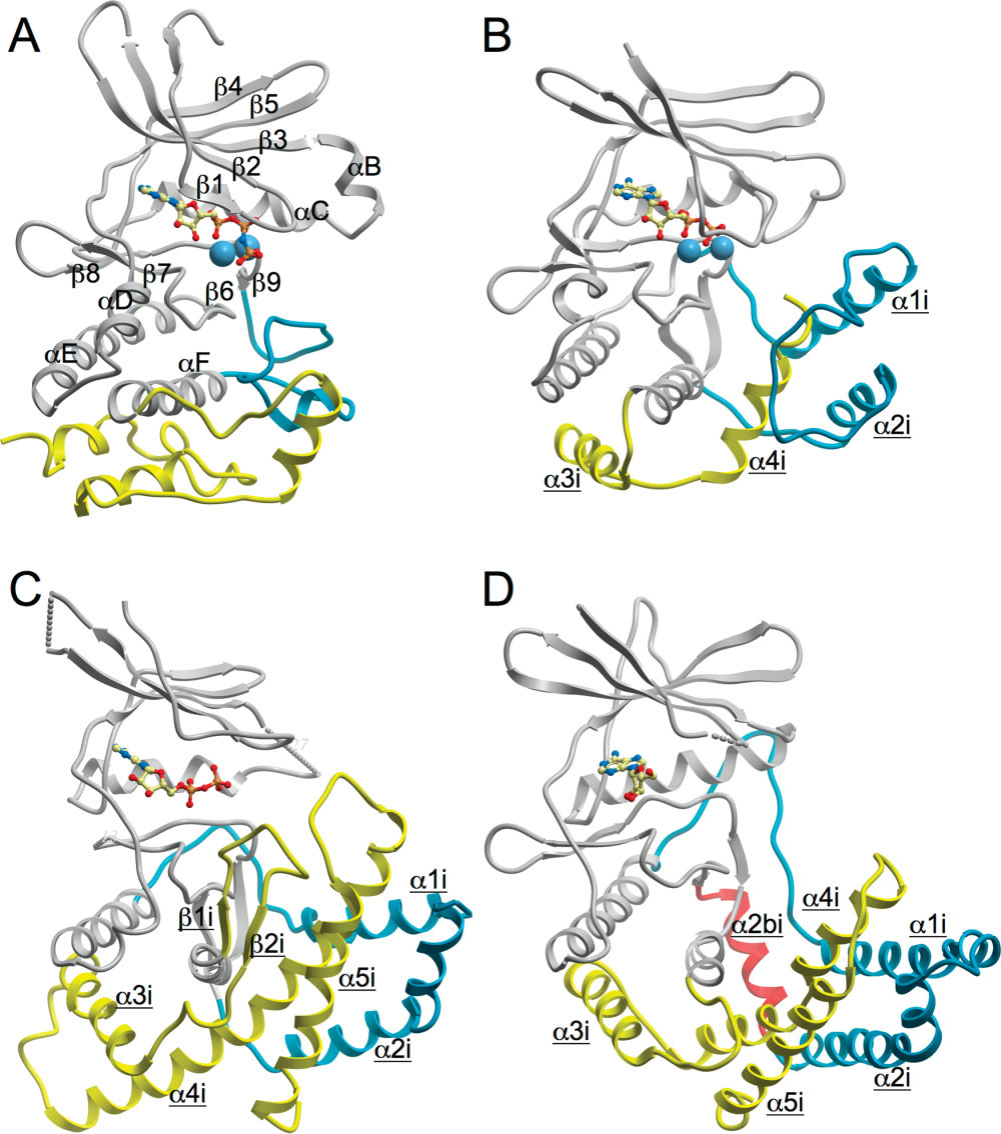
Overview comparison of YtaA with other CAKs and PKA. Common secondary structure elements shared by all structures are shown in gray, and labeled in the PKA structure. Distinctive structural elements specific to the CAKs are shown in blue and yellow, with the analogous (but structurally distinct) regions of the PKA structure shown in identical colors. The unique helix in YtaA, α2bi, is shown in red. The CAK-specific elements are labeled on all three CAK structures in underline. **A**: PKA; **B**: APH; **C**: ChoK; and **D**: YtaA.

Despite its overall similarity, YtaA is not closely related to any previously characterized CAK structures. The closest similarity is to homoserine kinase 2 (HSK2), an enzyme involved in threonine biosynthesis^59^ (Table III). Here, we compare the structure of YtaA to those of choline kinase (ChoK)^61^ and aminoglycoside phosphotransferase (APH),^50^ prototypical CAKs with well-characterized structures that also have a bound adenosine-derived cofactor, enabling comparison of the ATP binding sites. Of these two structures, YtaA is moderately more similar to ChoK (Table III). We also compare YtaA to a representative ePK, PKA.^62^ A substantial structural core, which encompasses the essential residues for ATP binding and phosphotransfer, is shared between ePKs and CAKs (see Fig. 4). We name shared structural elements by ePK conventions,^63^ and use an “i” suffix to label elements unique to CAKs.

**Table III:** Superposition of YtaA with Representative Structures from a Search with the Dali Server^60^

Structure	Classification	PDB ID: chain	Dali *Z*-score	RMSD (Å)	Aligned positions	%ID
Homoserine kinase 2	CAK	*2PPQ:A*	23.5	3.2	287	16
Choline/ethanolamine kinase	CAK	3DXQ:A	18.9	3.9	265	13
YihE	CAK	1ZYL:A	18.4	4.1	273	14
Choline kinase α-2 (ChoK)	CAK	*1CKP:A*	15.6	3.9	259	14
Methylthioribose kinase (MTRK)	CAK	2PUN:A	15.1	4.0	260	12
Aminoglycoside phosphotransferase (APH)	CAK	*1L8T:A*	13.1	5.1	219	12
RIO1	Rio	1ZTH:C	9.9	3.6	168	15
Protein kinase A (PKA)	ePK	*1CDK:A*	7.7	3.4	162	14

Structures with IDs in italics were used in analysis and comparisons with YtaA.

YtaA retains two CAK-specific structural elements in the C-terminal lobe^12^,^64^ (see Fig. 4). First, α1i-α2i is a large helical insertion [blue in Fig. 4(B–D)] after αE, which spatially replaces the ePK-specific activation segment^65^ [blue in Fig. 4(A)]. YtaA adds a new helix to the end of this insertion [α2bi, red in Fig. 4(D)]. Second, distinctive helices at the C-terminus join with the insertion to form a putative substrate binding site [α3i–α5i, yellow in Fig. 4(B–D)]. In YtaA, the number and position of these helices is more similar to that seen in ChoK than APH. The observed structural similarity in the C-terminal lobe of YtaA and the other CAKs suggests that YtaA uses this region to bind small molecule substrates, as was previously shown for ChoK^61^ and APH.^50^ While all three CAKs retain these distinctive elements, the structural similarity is lowest in these areas, with different number and placement of elements. It is likely that these differences at least partially reflect changes in the substrate specificity of the three enzymes.

### YtaA binds adenosine in a distinctive manner

The YtaA structure reveals a distinctive ATP binding pocket that is broadly similar to other CAKs, but has key elements that help to define BSKs as a distinct family. In some aspects, the YtaA pocket is more like that of ChoK, but in others it is more like the APH pocket.

**Figure 5:**
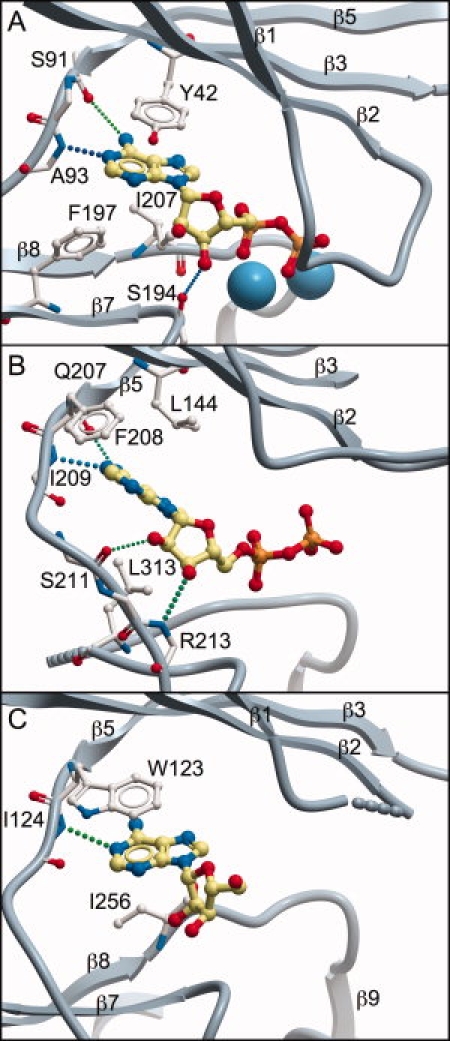
Comparison of the adenosine binding pocket in YtaA and other CAKs. Structures are presented in identical orientation, based on structural alignment and superposition with DaliLite.^30^ This presentation highlights changes in location and orientation of the adenosine molecule in each structure. Key adenosine-interacting residues in each structure are shown (side chains are omitted for residues that contribute only backbone atoms). H-bonds are shown as dotted lines and metal atoms as blue spheres. Portions of the structures are omitted to improve clarity. **A**: APH; **B**: ChoK; and **C**: YtaA.

In CAKs, the adenine ring of ATP usually interacts with another aromatic ring from the N-terminal lobe, but the specific interactions and the orientation of the ATP are different in each structure (see Fig. 5). This pattern is in contrast to ePKs, which have a stereotypical ATP binding mechanism: The primary hydrophobic interaction from the N-terminal lobe to the adenine ring of ATP is almost always via V57^PKA^ in β2 and A70^PKA^ in β3.^63^ In ChoK and YtaA, the interacting ring comes from the interlobe linker (W123^YtaA^ and F208^ChoK^). Although this side chain emanates from the same backbone location, in YtaA aromatic π-π stacking is observed, while in ChoK the rings interact in a perpendicular manner. As a result, in ChoK the face of the adenine ring also packs against L144^ChoK^ in β3 (see Fig. 5). In APH, the interacting ring instead comes from Y42^APH^ in β3 (corresponding to L144^ChoK^ and A70^PKA^), and stacks atop the adenine ring in a similar fashion to W123 in YtaA (see Fig. 5).^66^ In all three structures (as in PKA), the adenine ring also forms H-bonds to the protein backbone in the linker region. The changes in the interaction patterns produce substantial changes in the positioning and orientation of the adenosine moiety (see Fig. 5).

Residues forming the ATP binding pocket from the C-terminal lobe also vary between CAKs. ChoK forms a primary interaction from L313^ChoK^ in β7 and the ATP ribose also hydrogen bonds with the protein backbone in the linker region. YtaA instead forms the hydrophobic interaction with I256^YtaA^ from β8, and the ribose moiety forms no hydrogen bonds with the protein. APH uses both hydrophobic sites (F197^APH^ from β7 and I207^APH^ from β8). While the ribose of ATP still forms a hydrogen bond, it is to the backbone upstream from β7 (see Fig. 5).

Consideration of these three CAKs demonstrates that the ATP binding pockets are quite variable within the CAK family. It is possible, particularly in the case of YtaA, that the ATP molecule could shift position and orientation depending on the activation state of the enzyme and the binding of substrate.^61^ Only adenosine could be reliably modeled in the YtaA structure, and the ribose moiety is rotated into a position incompatible with proper placement of the phosphate groups for substrate phosphorylation (though the ribose appears partially disordered, see earlier). However, the two primary adenosine interacting residues, W123^YtaA^ and I256^YtaA^, are conserved in most putatively active BSKs, suggesting the observed interactions are both relevant and indicative of a family-wide pattern.

### The YtaA active site indicates it is likely to be a functional enzyme

**Figure 6:**
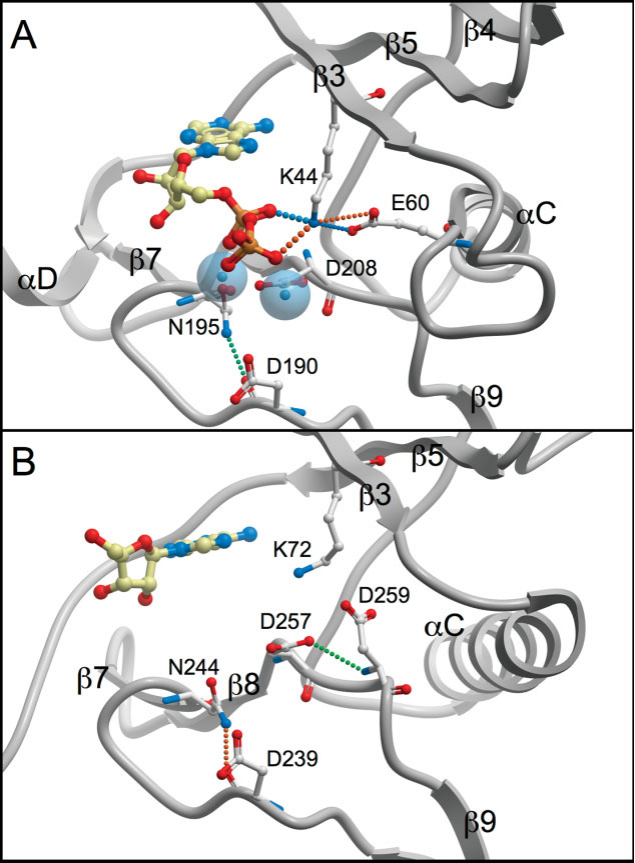
Comparison of active sites of APH and YtaA. Structures are presented in identical orientation, based on structural alignment and superposition with DaliLite.^30^ Conventions are as in Figure 5, except that metal atoms are shown as transparent spheres. **A**: APH and **B**: YtaA.

PKL kinases share a tightly integrated active site where the ATP phosphates are coordinated and positioned for optimal phosphotransfer. In ePKs, the K72^PKA^-E91^PKA^ ion pair links β3 and αC, while also providing a positive charge (K72) to interact with the negatively charged ATP phosphates. APH retains a similar K44^APH^-E60^APH^ ion pair, which fulfills a similar role in the APH structure (see Fig. 6). ChoK replaces the Lys residue with R146^ChoK^, a common substitution in CAKs.^11^

In contrast to other CAKs, YtaA retains the Lys (K72^YtaA^), but completely lacks the Glu partner, replacing it with S84^YtaA^ (see Fig. 1), which does not form an analogous interaction. The missing Glu may be functionally replaced by D259^YtaA^ in the Dxe motif (DLD in YtaA, Fig. 6). The side-chain carboxyl groups of D259^YtaA^ and E60^APH^ occupy the same spatial location (see Fig. 6), indicating that this sort of compensation is plausible. Further, D259 is often conserved as an acidic residue in CAKs^11^ and is highly conserved in most BSKs (see Fig. 1).

The remaining active site residues, which are required for metal binding and catalysis, are conserved in YtaA (as in APH and ChoK), indicating that this protein is very likely to be catalytically active. The three key residues D239^YtaA^ (D166^PKA^), N244^YtaA^ (N171^PKA^), and D257^YtaA^ (D184^PKA^), are in approximately standard conformations for a PKL kinase (see Fig. 6).

### Putatively active BSKs have a distinctive and highly conserved linker motif

**Figure 7:**
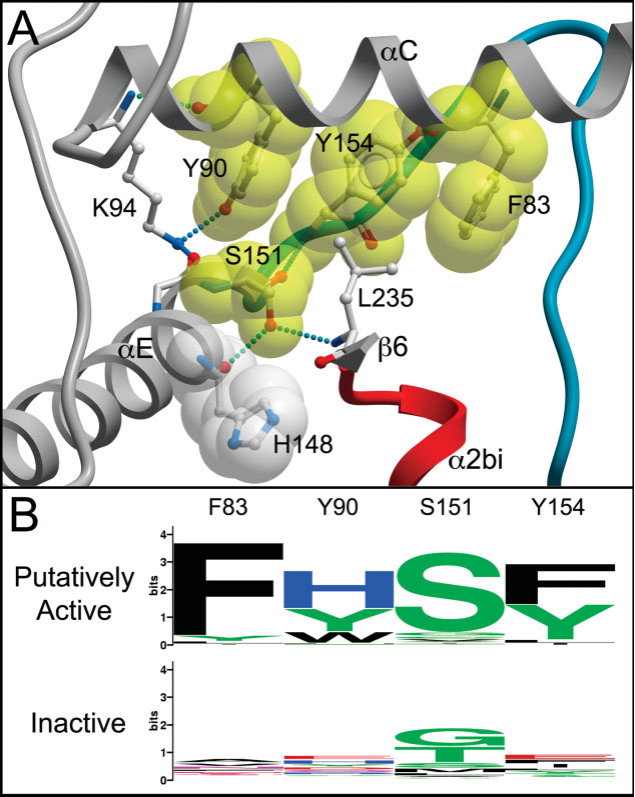
The hydrophobic linker motif conserved only in putatively active BSKs. **A**: Cutaway view of motif residue interactions. The YtaA structure is in approximately the same orientation as in Figure 4, with secondary structure elements identically colored. Motif residues are rendered with a yellow space-filling shell. H148, a residue highly conserved in almost all PKL kinases,^11^ is shown with a space-filling shell in white. Unconserved residues interacting with the motif are in ball-and-stick view. **B**: Logo of motif, showing selective conservation only in putatively active BSKs. The motif is discontinuous in sequence, and shown with intervening sequence removed.

Sequence comparison between putatively active and inactive BSKs reveals a highly conserved, mostly hydrophobic structural linker motif including F83^YtaA^, Y90^YtaA^, S151^YtaA^, and Y154^YtaA^, which is strongly associated with likely enzymatic activity (Fig. 1, hydrophobic linker section), though the motif does not directly interact with the active site (see Fig. 7). These conserved residues form a network of hydrophobic and H-bond interactions that link together αE, αC, β6, and the loop linking αE and α1i, which forms a convoluted structure along the “back” of the enzyme, opposite the active site (see Fig. 7). This motif effectively connects key portions of the two lobes. It also links to the hydrogen bond network that stabilizes the catalytic region of many PKL kinases (mentioned earlier) through an H-bond to H148^YtaA^. Given the strong correlation with conservation of catalytic motifs, and its linkage to known highly conserved residues, we propose that this motif is likely to stabilize the protein for proper enzymatic function.

The motif is also present, though not fully conserved, in HSK2 (see Fig. 1), but not other CAKs, further demonstrating the relatively close relationship between these two families. Interestingly, it is also present in CotS, suggesting that this enzyme may indeed be active, despite unusual sequence changes in some species.

### Conservation patterns in the putative BSK substrate binding site indicate a variety of distinct substrates

**Figure 8:**
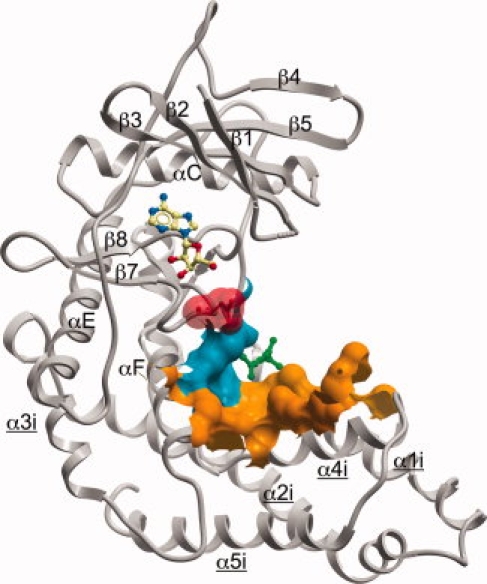
Predicted substrate binding region of the BSK family, mapped onto the YtaA surface. The orange and blue surface show the region generally conserved throughout the family. The blue region is specifically highly conserved in YtaA (Table IV). The key active site residue D239^YtaA^ is shown with a red space-filling shell. The unknown ligand in the YtaA structure (green) and the adenosine are shown in ball-and-stick.

Previous structures of CAKs bound to substrate have defined a substrate binding region incorporating residues from α1i–α2i, the catalytic loop, αF, and α4i–α5i.^50^,^61^ Evolutionary constraint analysis of the entire BSK family with ConSurf^37^ reveals a conserved surface region in this same location, defining the putative substrate binding site for BSKs (Table IV). This site forms a bowl-shaped pocket with a complex surface (see Fig. 8). Remarkably, the electron density maps for the YtaA structure show additional density for an unknown ligand in this region. However, it is unlikely that this ligand could be the biological substrate of YtaA: it is distant from the key active site residue D239^YtaA^ (D166^PKA^), which directly coordinates the substrate hydroxyl group.^49^

**Table IV:** The Conserved Putative Substrate Pocket of the BSK Family, Mapped to YtaA. Listed Positions Display Overall Conservation in the Family

YtaA residue	Secondary structure element
R176	α1i
Q179	α1i
Q238^a^	Catalytic loop
R268^a^	αF^a^
R271^a^	αF^a^
K272^a^	αF^a^
M273	αF
I275	αF
P276	αF
Y318	α4i
E319	α4i
Y321	α4i
D322	α4i
R325	α4i

a Highly conserved specifically in YtaA.

Although the substrate binding region is generally conserved throughout the BSKs, each group of orthologs in the family displays distinct subsets of highly conserved residues within the pocket (Fig. 1, putative substrate binding section). YtaA has four highly conserved residues, of which three are basic (Table IV), forming a patch directly adjacent to D239 (blue in Fig. 8). CotS has weaker conservation in this patch, but adds an additional region of higher conservation. Remarkably, YutH retains a highly conserved site, despite its catalytic inactivity, indicating that it is very likely to still bind a pseudosubstrate. By contrast, the YsxE binding site is very poorly conserved, and probably does not bind a ligand. This general pattern is repeated in *Clostridiales*, with BSKC6 having a particularly poorly conserved site, further emphasizing its rapid evolutionary degradation. This pattern strongly suggests that each protein in the family has distinct substrate binding properties. The equivalent residues in HSK2 also differ substantially, (see Fig. 1), indicating that BSKs are unlikely to be homoserine kinases.

## CONCLUSIONS

The BSKs are a new family of bacterial kinases with distinctive structural features and an unusual subcellular location, with most members packaged into the bacterial spore coat. Although the precise functions of BSKs are unknown, our integrated genomic, phylogenetic, and structural approach has highlighted several attributes of the family.

The dynamic evolutionary pattern seen in BSKs suggests that they provide multiple specific functional enhancements to different species, rather than acting as core structural elements of the coat. This notion is supported by the absence of BSKs from some sporulating species, such as *C*. *difficile*, and the mild phenotypes of BSK mutants.^7^,^8^,^45^ Further diversity comes from frequent apparent loss of catalytic activity, suggesting that BSKs have a common, and perhaps predominant, nonenzymatic function. The diverse sequence conservation patterns in the putative substrate binding site also suggest distinct functions, and indicate that some BSK pseudokinases could function through binding pseudosubstrates. The tight association between some BSKs and glycosyl transferases and predicted nucleotide sugar metabolizing enzymes suggests that they may bind or phosphorylate one of these reactants, correlating with the aminoglycoside substrates of the related APHs. Given the relevance of spore formation to bacterial pathogenesis, and the demonstrated drugability of PKL kinases,^67^,^68^ we believe further experimental characterization of this family is warranted.

The structure of YtaA also illuminates the remarkable innovations that have occurred in the active site of CAK kinases. Although the ePK family is very diverse, the mode of ATP binding and the conservation of active site residues is almost identical across the entire family, with only a few narrow exceptions.^69^,^70^ In contrast, CAKs often display substantial changes to the residues in the ATP binding pocket and catalytic region.^11^ Our comparison of YtaA with the structures of other CAKs reveals the substantial degree of structural variability coincident with these sequence changes. Consideration of the broader PKL superfamily has revealed a wide range of structural changes in substrate binding regions,^12^ and the CAKs now demonstrate that such changes can even propagate into the active sites. The variability in CAK active sites may be due to the wide variety of molecules that they must phosphorylate: each CAK may be enzymatically optimized for its specific substrate.^71^ In contrast, while the ePKs display a broad range of peptide motif specificity,^72^ the ultimate catalytic target of these enzymes is much more restricted: the hydroxyl groups of serine, threonine, and tyrosine residues. Thus, as with YtaA, new structures of CAKs should continue to provide insights into the true catalytic plasticity of the PKL fold.
